# Field applications of zein as a precise nanoscale delivery system for methoxyfenozide

**DOI:** 10.1093/jisesa/iead017

**Published:** 2023-03-24

**Authors:** C A R Bonser, C Tamez, J C White, C E Astete, C M Sabliov, J A Davis

**Affiliations:** Department of Entomology, LSU Agricultural Center, 404 Life Science Building, Baton Rouge, LA 70803, USA; Connecticut Agricultural Experiment Station, New Haven, CT 06511, USA; Connecticut Agricultural Experiment Station, New Haven, CT 06511, USA; Department of Biological and Agricultural Engineering, LSU Agricultural Center, 149 E. B. Doran Building, Baton Rouge, LA 70803, USA; Department of Biological and Agricultural Engineering, LSU Agricultural Center, 149 E. B. Doran Building, Baton Rouge, LA 70803, USA; Department of Entomology, LSU Agricultural Center, 404 Life Science Building, Baton Rouge, LA 70803, USA

**Keywords:** soybean, insecticide residual, foliar application, biopolymer nanoparticle

## Abstract

When insecticides are applied in the environment, much of the product does not reach the target pest. Biopolymeric nanoparticles as nanocarriers have the potential to improve insecticide efficacy by improving absorption, coverage, and permeability while protecting the insecticide active ingredient from abiotic conditions and extending efficacy through controlled release. We conducted a series of experiments using a biopolymeric nanoparticle synthesized from zein, a biodegradable maize protein, to compare efficacy of a nanodelivered hydrophobic insect growth regulator (methoxyfenozide) against a commercial formulation. Positively charged zein nanoparticles (empty and loaded with methoxyfenozide) were compared to the formulated product, Intrepid 2F, as a foliar spray in-field on soybean. *Chrysodeixis includens* (Walker) was used as a model and was fed sprayed soybean leaves to evaluate efficacy of the tested foliar products over time. A separate set of leaves was sampled to measure residue levels of methoxyfenozide (MFZ) over time following foliar application using QuEChERS extraction and high-resolution liquid chromatography-mass spectrometry. Regression analysis found no differences in mortality slopes between positively charged zein nanoparticles loaded with methoxyfenozide [(+)ZNP(MFZ)] and Intrepid 2F, suggesting comparable efficacy of the synthesized nanoparticles to a commercial product. Higher concentrations of MFZ were present in (+)ZNP(MFZ)-treated in leaf tissue at 3 d following spray when compared to Intrepid 2F. The multiyear study results demonstrate that nanoparticles loaded with MFZ are comparable to Intrepid 2F under field conditions, with potential short-term benefits.

When insecticides are applied to plants, much of the product does not reach the intended target pest (Pimentel 1995, Wang et al. 2022). Nanoparticles as nanocarriers have the potential to increase insecticide delivery by improving or controlling absorption, coverage, and permeability while protecting insecticides from abiotic conditions and extending efficacy through slow release (Hou et al. 2021). Nanoparticles are agglomerations of materials, normally in the range of 1–100 nm, with increased surface area to volume ratios, resulting in functional characteristics divergent from their bulk counterparts (Auffan et al. 2009). Thus, nanoparticles could improve insecticide applications and reduce environmental pollution by delayed or even tunable release of active ingredients and selective targeting.

Most agricultural nanoparticle research is focused on the use of inorganic nanoparticles (Nel et al. 2006, Salinas et al. 2021), and although these materials show promise in agriculture, concerns regarding unintended consequences exist, such as the accumulation of particles or ions in select environmental and biotic compartments, as well as a potential enhanced ability to penetrate cellular membranes (Ahamed et al. 2008, Nowack 2009, Navarro et al. 2008, Yin et al. 2022, Węsierska et al. 2018). Environmentally safe and sustainable replacements for metal nanoparticles are needed and biopolymer nanoparticles have attracted increasing interest. Biopolymer nanoparticles are degradable (Vroman and Tighzert 2009) and have been evaluated as successful insecticide delivery agents in laboratory conditions (de Oliviera et al. 2018, Bonser et al. 2022, Mendez et al. 2022). However, the impact of these nanoparticles in delivering agrochemicals is rarely evaluated in field studies.

To address these deficiencies, an experiment evaluating the biopolymer zein, a protein extracted from maize, as a nanodelivery system for methoxyfenozide (MFZ) was conducted. Previous research has suggested that positively charged zein nanoparticles with entrapped MFZ [(+)ZNP(MFZ)] may have greater deleterious effects on soybean lepidopteran defoliators than MFZ (Bonser et al. 2022). However, those trials were conducted under laboratory conditions and field applications of (+)ZNP(MFZ) have yet to be conducted in order to demonstrate future applicability. Thus, formulations of (+)ZNP(MFZ) were compared to a formulated commercial insecticide with the same active ingredient, Intrepid 2F at equivalent MFZ concentrations. Efficacy over time was evaluated under field conditions by sampling soybean [*Glycine max* (L.) Merr.] leaf tissue at different intervals post-spraying and feeding them to the soybean looper, *Chrysodeixis includens* (Walker). To evaluate adsorption and coverage, residual levels of MFZ were measured at different time points. This study increases understanding of the use of novel nanoscale biopolymer carriers as part of sustainable precision agriculture.

## Materials and Methods

### Soybean Looper Insect Colony Maintenance

*Chrysodeixis includens* (Walker), LSU1, originally collected from south Louisiana in 1976 (Newsom et al. 1980), was selected as a model insect to test the efficacy of the nanodelivered MFZ. The colony was maintained as described in Bonser et al. (2022). In brief, newly eclosed neonate larvae were placed into 30 ml Solo soufflé cups (Dart Container Corporation, Mason, MI, USA) on a meridic diet (Southland Products Inc., Lake Village, AR, USA) prepared as per manufacturer’s instructions. Cups were placed into a rearing room at 26°C with 50% RH and 14:10 (L:D) photophase. Once the insects reached the pupal stage, individuals were removed from the cups and placed onto a bed of vermiculite (Sungro Horticulture, Bellevue, WA, USA) at the bottom of 5.7-liter round food storage containers (Parade Plastics, Mooresville, IN, USA). Containers were lined with brown single-fold paper towels (25.5 × 7.5 cm; Georgia Pacific, Atlanta, GA, USA) as an oviposition substrate. Adults were fed a solution of 90:10 distilled water–honey solution placed in a 30 ml cup filled with cotton wadding (Barkman Honey, LLC, Hillsboro, KS; Medline Industries, Inc., Mundelein, IL, USA). Egg sheets were collected and placed into bags every 2 d, whereby larvae eclosed and the process was restarted.

### Preparation of Biopolymeric Nanoparticle Formulations

The preparation of zein nanoparticles for foliar spray followed methodology described in Bonser et al. (2022). Empty and MFZ loaded positively charged zein nanoparticles [(+)ZNP and (+)ZNP(MFZ)] were formed by an emulsion-diffusion method. The organic phase was first prepared by dissolving 1,100 mg of zein powder in 30 ml of a 4:1 (v/v) acetone/water solution. The organic phase was added to the aqueous phase, which consisted of 300 ml of low resistivity water (Thermo Fisher Scientific, Waltham, MA, USA) and 173 mg of didodecyldimethylammonium bromide (DDAB) (MilliporeSigma, St. Louis, MO, USA). The emulsion was run through a microfluidizer (M-110P; Microfluidics International Co., Westwood, MA, USA) thrice at high pressure (172,000 KPa) prior to solvent evaporation. The acetone was evaporated using a rotary evaporator (Rotavapor R-300; BÜCHI Labortechnik, New Castle, DE, USA) under vacuum at 33°C for 60 min. The zein nanoparticles loaded with MFZ were synthesized by the same protocol with the addition of 100 mg of MFZ (Fisher Thermo Scientific, St. Louis, MO, USA) in the organic phase. The resulting zein biopolymer nanoparticles used in all experiments were in a suspension.

### Characterization of Biopolymeric Nanoparticle Formulations

Characterization of (+)ZNP and (+)ZNP(MFZ) was performed by using dynamic light scattering (DLS) Malvern zetasizer (Malvern Panalytical Ltd, Southborough, MA, USA) at 25°C using a monomodal distribution. The values of size, size distribution, and zeta potential reported were the mean of three independent replicates. The samples were diluted with high resistivity water at pH 6. The transmission electron microscopy (TEM) JEOL 1400 series (Jeol USA Inc., Peabody, MA, USA) with an accelerating voltage of 120 kV was used to study zein nanoparticles morphology and 1% uranyl acetate (ThermoFisher Scientific Inc., Pittsburgh, PA, USA) was added as contrast agent before placing a sample droplet over the copper grid.

### Field Design and Planting

Soybean test plots were planted in Baton Rouge, Louisiana, USA at the Doyle Chambers Central Research Station (30.368°N, 91.1665°W) from 2020 to 2022. Cultivars, treatment dates, and sampled stages for each year are detailed in Table 1. Seeds were planted at eight seeds per 0.30 m on 0.76 m centers using a four-row cone planter (Almaco, Nevada, IA, USA). Test plots consisted of either two rows (2020) or one row (2021–2022) at 9.14 m × 0.76 m. Plots were arranged in a randomized block design with four replicates (*n* = 16). No insecticides were applied except for the treatments and all plots were managed according to best agronomic practices for the area (Moseley et al. 2022).

**Table 1.** Field replications with cultivars and treatment dates

Rep	Year	Cultivar	Stage	Treatment date	Planting date
1	2020	Asgrow 46X0	R2	03 Jun	20 Apr
2	2020	Asgrow 46X0	R5	20 Jun	20 Apr
3	2020	Asgrow 53X0	R5	11 Aug	22 May
4	2021	Asgrow 46X0	R2	10 Jun	23 Apr
5	2021	Armor 46D08	R2	15 Jun	25 May
6	2021	Asgrow 46X0	R5	30 Jul	23 Apr
7	2021	Armor 46D08	R5	31 Aug	25 May
8	2022	Asgrow 43F0	R2	27 Jun	23 Apr
9	2022	Asgrow 43F0	R2	15 Jul	23 May
10	2022	Asgrow 43F0	R5	20 Jul	23 Apr

Soybean stage R2 refers to full flower bloom stage in soybean and R5 refers to the beginning seed formation stage.

### Foliar Application

Each plot was sprayed with 125 ml of treatment using a CO_2_ backpack sprayer (R&D Sprayers, Opelousas, LA, USA) calibrated to deliver 140.2 l/ha at 241.3 kPa using four TeeJet 80015VS flat-fan nozzles (TeeJet Technologies, Springfield, IL, USA). The sprayer equipment and nozzles were thoroughly rinsed with 1,000 ml of water before and after spraying each treatment formulation.

### Methoxyfenozide Efficacy Bioassay

In order to determine if nanocarried MFZ by (+)ZNP(MFZ) was as effective as a commercially available MFZ insecticide, a leaf tissue bioassay using *C*. *includens* as a model was conducted. Plants were sprayed as described above with one of the four following treatments: distilled water (untreated check), (+)ZNP at 2,320 ppm, (+)ZNP(MFZ) at 2,320 ppm zein and 200 ppm MFZ, and Intrepid 2F (Corteva Agriscience, Indianapolis, IN, USA) at 200 ppm. Fresh nanoparticles were synthesized each time prior to spraying; solutions of Intrepid 2F were also made immediately prior to use. Soybean plants were sprayed at both full bloom (R2) and pod fill (R5) stages (Fehr and Caviness 1977). Soybean leaf tissue was then sampled at five different points: before spraying, 1 d after, 3 d after, 7 d after, and 14 d after treatment (DAT). This allowed us to determine efficacy over time under natural weathering conditions. Over the span of three years (2020–2022), the experiment was replicated ten separate times at different points within the growing seasons (Table 1). Each replicate was concluded at 14 DAT, when no differences were found between treatments.

Sampled soybean leaves were picked from the top of the row, with 15 random leaves sampled from each treatment within a block at each time point. Leaves were immediately brought back to the laboratory, cored using 11.34 cm^2^ #149 Arch Punch (Osborne and Co. Harrison, NJ, USA), and placed into a sterile Petri dish (100 × 15 mm) (Fisherbrand Petri Dishes, ThermoFisher Scientific Inc., Pittsburgh, PA, USA) lined with dampened qualitative filter paper 410 (9.0 cm dia.) (VWR International, Radnor, PA, USA). Individual neonates of *C*. *includens* were transferred to leaf cores, one per core, using a nylon paintbrush (Royal Langnickel Gold Brushes, Royal Brush Manufacturing, Munster, IN). Petri dishes with their respective leaf cores and neonates were placed into the rearing room with conditions of 26°C, 50% RH, and 14:10 (L:D). Petri dish filter paper was checked and moistened daily. Larval weights, mortality, and leaf defoliation were assessed 7 d following placement. Larval weights were measured using a Mettler-Toledo XS105 scale (Mettler-Toledo, Columbus, OH, USA). Leaf defoliation was estimated visually to increments of 5% using the leaf defoliation chart by Ortega et al. (2016) as a guide. Larvae were considered dead following the failure of an individual to respond to prodding using a nylon paintbrush.

### Methoxyfenozide Residue Analysis

To measure the effect of nanoscale carrier on the residue retention of MFZ, leaf tissue was harvested at 3 d and 7 d following foliar application of the previously mentioned nanoparticle treatments in 2021. Leaf tissue in this experiment was collected only from plants that were sprayed with the control, (+)ZNP(MFZ), and Intrepid 2F; empty (+)ZNP treatments were excluded from testing as they were not carriers of MFZ. Four blocks were sampled per spray period, with four different sprays conducted (*n* = 16). Thirty random leaves were collected from each treatment replicate, placed into plastic bags, and stored at −80°C. To analyze residue concentrations, a modified QuEChERS procedure (Quick, Easy, Cheap, Effective, Rugged, and Safe [Lehotay et al. 2005]) was used for extraction from the collected leaf tissue. Leaf tissue samples were put on dry ice and shipped overnight to the Connecticut Agricultural Experiment Station (New Haven, CT, USA) in October 2021, the site of QuEChERS extractions and chemical analysis.

Extractions were conducted by taking 5.0 g of thawed leaf tissue and finely mincing it before transferring it to a 50.0 ml polypropylene centrifuge tube with 15.0 ml of distilled water and 15.0 ml acetonitrile (MeCN). Tubes were gently shaken by hand before adding QuEChERS salts (6.0 g of MgSO_4_ and 1.5 g of NaOAc), whereby the tubes were shaken vigorously using a paint can shaker for 30 min and centrifuged for 10 min at 3,000 RPM. Supernatant samples were stored in the freezer at −20°C until analysis. Aliquots for each supernatant were analyzed by liquid chromatography-mass spectrometry using a Dionex UltiMate 3000 liquid chromatograph interfaced with a Thermo Velos Pro mass spectrometer (Thermo Fisher Scientific, Waltham, MA, USA) equipped with an Agilent SB-C18-RRHD-2.1 mm × 150 mm column packed with 1.8 μm particles (Agilent Technologies, Santa Clara, CA, USA). Calibration curves were obtained by making a series of MFZ standards at different concentrations. A seven point external standard calibration curve was used with continuing calibration verification injections performed at the beginning, throughout, and at the end of each analytical batch. For quality assurance, every analytical run contained a reagent blank and continuing calibration verification (CCV) samples at regular intervals. Each extraction batch contained a method blank and a spike recovery sample. Extractions and standards were prepared gravimetrically.

### Data Analyses

All data were analyzed in R 4.2.1 using IDE RStudio (RStudio Team 2022, R Core Team 2022) or Microsoft Excel version 2010 (Microsoft Corporation 2010). To test the effect of foliar applied nanoparticle solutions on leaf consumption, a repeated-measures analysis of variance (ANOVA) was used to compare larval weights and leaf defoliation with treatment, time, and the interaction between treatment and time; designated as fixed effects. Year was treated as a random effect in the model. Comparisons between DAT were made using R package *emmeans* with an adjusted Tukey comparison (α = 0.05) (Lenth 2022). Proportion data for leaf defoliation were arcsine-square root transformed to achieve normality using the Shapiro–Wilks test. A linear regression model was used to assess the differences in mortality proportions with respect to days after treatment of nanoparticle solutions using package *lme4* (Bates et al. 2015). Pairwise *t*-tests were used to compare the slopes between treatments. Data from MFZ chemical analysis was corrected using the Schneider-Orelli formula (Pünterner 1981) and was square-root transformed to achieve normality of residuals. A two-way ANOVA using R package *lme4* compared the treatments to DAT, with replicate treated as a random factor. A Pearson’s Correlation was conducted to account for late summer rainfall in the Louisiana 2021 growing season at the Doyle Chambers Central Research Station and the variation in extracted MFZ in replicates of the QuEChERS extraction. Rainfall data was downloaded from the Louisiana Agriclimatic Information System <https://weather.lsuagcenter.com/>.

## Results

### Nanoparticle Characterization

**Fig. 1.**
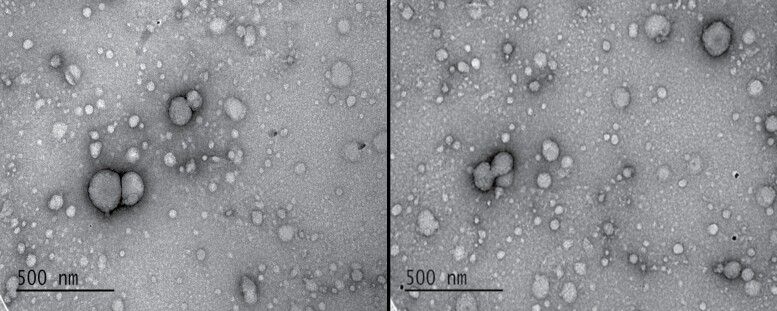
Images of zein nanoparticles using transmission electron microscope. The image on the left is of empty positively charged zein nanoparticles [(+)ZNP], while the image on the right is of positively charged zein nanoparticles loaded with methoxyfenozide [(+)ZNP(MFZ)].

Transmission electron microscopy images of zein biopolymer nanoparticles used are shown in Fig. 1. Based on dynamic light scattering (DLS) analysis, (+)ZNP(MFZ) had a hydrodynamic diameter of 136.0 ± 9.5 nm, and a polydispersity index (PDI) of 0.187 ± 0.034, indicating a monodisperse sample. The loaded biopolymeric nanoparticles exhibited a ζ-potential of +42.1 ± 7.3 mV. The empty (+)ZNP hydrodynamic diameter was 126.0 ± 6.9 nm and the PDI was 0.147 ± 0.019, again indicating a monodisperse sample. The particles carried a positive ζ-potential charge of +56.6 ± 4.7 mV.

### Methoxyfenozide Efficacy Bioassay

**Fig. 2.**
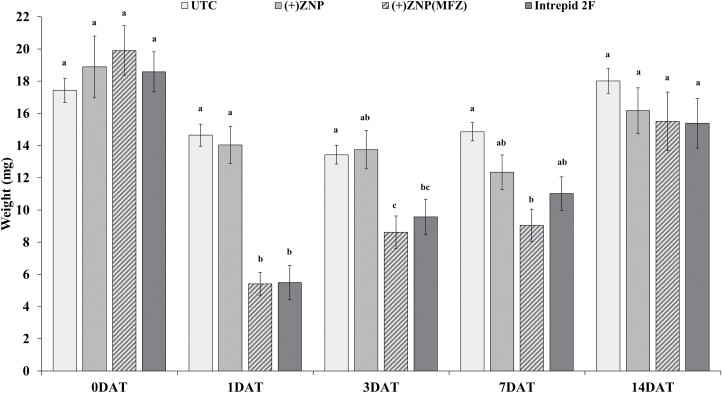
Mean (±SE) weights (mg) of *Chrysodeixis includens* following 7 d consumption of foliar treated nanoparticle leaf tissue at different days after treatment (DAT). Within a DAT, bars with the same letters are not significantly different (*P* < 0.05). UTC = control, (+)ZNP = zein nanoparticles, (+)ZNP(MFZ) = zein nanoparticles with nanoentrapped methoxyfenoxide.

The repeated-measures ANOVA found statistical differences in larval weights between the treatments (*F *= 12.9; df = 3, 680; *P* < 0.001), DAT (*F *= 36.5; df = 4, 680; *P* < 0.001), and the interaction between treatment and DAT (*F *= 3.5; df = 12, 680; *P* < 0.001) (Fig. 2). Weights of *C*. *includens* at 1 DAT were found to be reduced by 61% in both (+)ZNP(MFZ) and Intrepid 2F compared to both the control and (+)ZNP. At 3 DAT, larval weights continued to be reduced by 37% in (+)ZNP(MFZ) when compared to the control and (+)ZNP, but reductions were only 30% in Intrepid 2F—although values were not significantly different from each other. At 7 DAT, no significant differences in weights between (+)ZNP(MFZ) and Intrepid 2F were detected, though (+)ZNP(MFZ) reduced weights by a third, and Intrepid 2F reduced weights by a quarter.

**Fig. 3.**
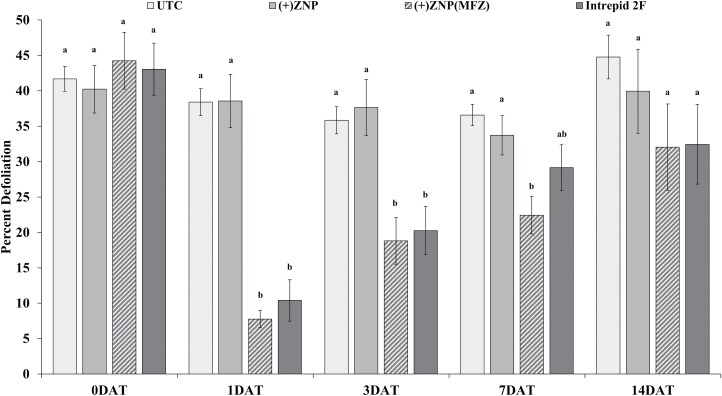
Mean (±SE) percent defoliation of soybean leaves by *Chrysodeixis includens* following 7 d consumption of foliar treated nanoparticle leaf tissue at different days after treatment (DAT). Within a DAT, bars with the same letters are not significantly different (*P* < 0.05). UTC = control, (+)ZNP = zein nanoparticles, (+)ZNP(MFZ) = zein nanoparticles with nanoentrapped methoxyfenoxide.

A repeated-measures ANOVA found statistical differences in leaf defoliation amongst insects between the treatments (*F *= 39.1; df = 3, 699; *P* < 0.001), DAT (*F *= 30.2 df = 4, 699; *P* < 0.001), and the interaction between treatment and DAT (*F *= 7.2; df = 12, 699; *P* < 0.001) (Fig. 3). Average defoliation across all DAT for the control and (+)ZNP treated soybean leaves was 40%. Leaf defoliation of (+)ZNP(MFZ) and Intrepid 2F soybean was 9%, a reduction of ~75% when compared to the control and (+)ZNP at 1 DAT. Although no significant differences in leaf defoliation between (+)ZNP(MFZ) and Intrepid 2F were detected in any DAT, (+)ZNP(MFZ) carried greater reductions when compared to the control and (+)ZNP (Fig. 3). For example, at 7 DAT, the leaf defoliation percentage for Intrepid 2F was decreased by a fifth when compared to the control and (+)ZNP, whereas it was reduced by a third for (+)ZNP(MFZ)-treated leave tissue.

**Fig. 4.**
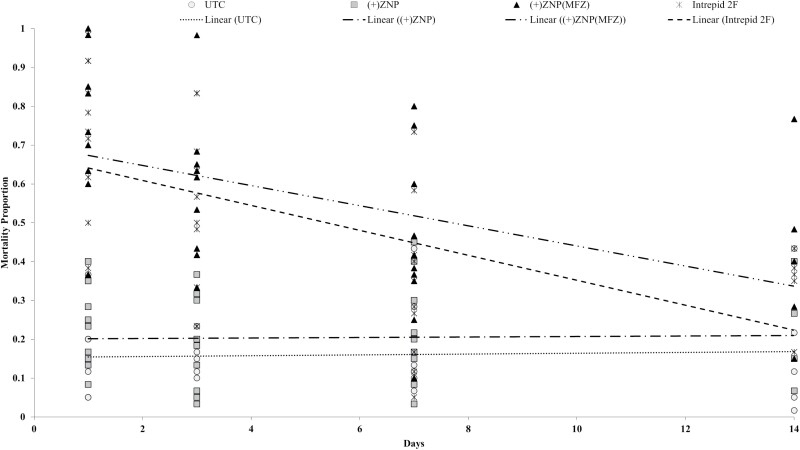
Regression chart demonstrating the relationship between days after treatment (DAT) of foliar application of formulations and *Chrysodeixis includens* mortality proportions. UTC = control, (+)ZNP = zein nanoparticles, (+)ZNP(MFZ) = zein nanoparticles with nanoentrapped methoxyfenoxide.

A linear regression was used to compare the relationship between mortality to days after treatment (DAT) for each treatment (Fig. 4). A significant interaction was found in the relationships of mortality percentages to DAT (*F *= 5.8; df = 3, 132; *P* < 0.001). The slopes for each of the treatments were as follows: control (slope = −0.00*x + 0.15; *R*^*2*^ = 0.00), (+)ZNP (slope = 0.00*x + 0.20; *R*^*2*^ = 0.00), (+)ZNP(MFZ) (slope = −0.03*x + 0.70; *R*^*2*^ = 0.23), and Intrepid 2F (slope = −0.03*x + 0.67; *R*^*2*^ = 0.31). Significant differences in slopes were found between control and (+)ZNP(MFZ) (*t* = 2.66; df = 66; *P* = 0.010), control and Intrepid (*t* = 3.19; df = 66; *P* = 0.002), (+)ZNP and (+)ZNP(MFZ) (*t* = 2.68; df = 66; *P* = 0.010), and (+)ZNP and Intrepid 2F (*t* = 3.19; df = 66; *P* = 0.002). Differences were not detected in slope comparisons between control and (+)ZNP, and between (+)ZNP(MFZ) and Intrepid 2F.

### Methoxyfenozide Residue Analysis

**Fig. 5.**
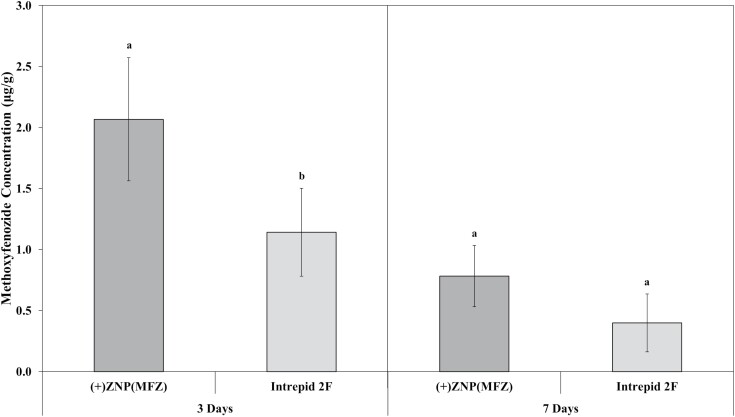
Mean (±SE) concentration of methoxyfenozide in sampled leaf tissue. The interaction between DAT and treatment (*F* = 5.2; df = 2, 87; *P* < 0.01) was found to be significantly different. Values were corrected using Schneider-Orelli’s formula, removing control values from the graph. Within a day, bars with the same letters are not significantly different (*P* < 0.05).

Chemical analysis of leaf tissue for MFZ demonstrated significant differences between the treatments (*F* = 56.7; df = 2, 87; *P* < 0.010) and in the interaction between DAT and treatment (*F* = 5.2, df = 2, 87; *P* < 0.010) (Fig. 5). A post hoc (*P* < 0.05) test revealed that sampled leaf tissue had a higher concentration of MFZ in (+)ZNP(MFZ) at 3 DAT than present in Intrepid 2F treated leaves. At 3 DAT, average concentrations of MFZ were 2.07 μg/g and 1.14 μg/g for (+)ZNP(MFZ) and Intrepid 2F, respectively. No differences were detected between treatments at 7 DAT, with the average MFZ concentrations 0.78 μg/g and 0.40 μg/g for (+)ZNP(MFZ) and Intrepid 2F, respectively. Differences were noted between replicates (*F* = 56.7; df = 2, 87; *P* < 0.010), with the third replication having a 95% higher concentration of MFZ present than the fourth replication. The Pearson’s Correlation that accounted for late summer rainfall in the Louisiana 2021 soybean growing season found a significant correlation between residual MFZ and rainfall at 7 DAT for (+)ZNP(MFZ) (*r* = −0.86; df = 14; *P* = 0.010).

## Discussion

The presented research evaluated the efficacy of methoxyfenozide as a model active ingredient when encapsulated by zein biopolymeric nanoparticles and is one of the first to document how nano-entrapped methoxyfenozide is affected following field exposure. Methoxyfenozide is a diacylhydrazine insecticide that acts as an ecdysone agonist on 20-hydroxyecdysone receptors in Lepidoptera (Wing et al. 1988, Palli and Retnakaran 2001, Doucet et al. 2009). Methoxyfenozide has been reported to have very low toxicity to humans (CAT IV) and generally minimal amounts entering aquatic organisms due to its poor solubility in water (U.S. EPA 2015, Chen et al. 2021). Because of this specificity, methoxyfenozide was selected as a model insecticide for evaluation. Bonser et al. (2022) demonstrated that nanoencapsulated methoxyfenozide in zein was more effective than the active ingredient alone on *C*. *includens*. *Chrysodeixis includens* reared on diet treated with 0.027 ppm (+)ZNP(MFZ) experienced prolonged pupal duration to adulthood (40%) compared to neat analyte MFZ and exhibited the lowest values of adult egg deposition and egg eclosion rates (Bonser et al. 2022). The former laboratory results indicate greater efficacy of the nano-enabled formulation and highlight the need for field evaluation of (+)ZNP(MFZ).

Application of (+)ZNP(MFZ) to leaf tissue was used to determine active ingredient longevity under field conditions when compared to a formulated product. Regression analysis suggests that at 14 DAT, no differences between the treatments and control exist. The concentrations of Intrepid 2F and MFZ (200 ppm) used in this study were significantly lower than what would be used under normal growing conditions. The labeled rates for Intrepid 2F applied to soybean are 292–585 ml/ha for *C*. *includens*, which equates to 600–1,200 ppm/ha. Research evaluating Intrepid 2F residual efficacy found that on leaf tissue the product can last in the field for approximately 14 days (Davis and Richter 2011). The research from this study indicates that (+)ZNP(MFZ) are as efficacious as Intrepid 2F at equal concentrations of active ingredient, demonstrating that nanoparticles have the potential to be effective carriers of insecticides in the field.

Biopolymer nanoparticles as nanocarriers can serve as a protective reservoir for active ingredients, which can be controlled depending on degradation and permeability of the synthesized polymer (Athanassiou et al. 2018). Zein nanoparticles have been shown to protect the entrapped active ingredient against abiotic factors, such as radiation from ultraviolet light. Research by de Oliveira et al. (2018) showed that geraniol, when encapsulated in zein nanoparticles, had a 17-fold decrease in degradation by ultraviolet radiation compared to the emulsified control. Zein nanoparticles were also found to protect lutein, a carotenoid, against ultraviolet degradation than that of the control emulsion (Chuacharoen and Sabliov 2016). de Oliveira et al. (2018) attributes the increased protection against ultraviolet radiation and chemical stability to the presence of aromatic amino acids in the structure of the zein protein. Results from this study also suggest that zein nanoparticles provide protection against the sun, as evidenced by the higher concentrations of MFZ residuals found in (+)ZNP(MFZ)-treated leaves at 3 DAT.

However, the results of the field application presented here do not agree with laboratory dialysis research conducted on zein nanoparticles by Hanna et al. (2022). A release profile of negatively charged zein biopolymer nanoparticles with methoxyfenozide [(−)ZNP(MFZ)] was performed by Hanna et al. (2022) to aid in the assessment of entrapped MFZ to free MFZ. Release rate constants of entrapped MFZ at 200 ppm were found to be 0.18 h^−1^, indicating that all nano-entrapped MFZ would be free within a 24 h period (Hanna et al. 2022). This would suggest that at 3 DAT, all MFZ in the (+)ZNP(MFZ)-treated leaves would no longer be bound; however, results indicate greater residual concentrations in leaves treated nano-entrapped formulation than in Intrepid 2F. Because free MFZ would be expected degrade in the presence of environmental exposure, this would suggest that the MFZ entrapped within (+)ZNP(MFZ) is not releasing as quickly as it would in dialysis. The discrepancy between this research and Hanna et al. (2022) could be due to the difference in nanoparticle charges tested or the different media tested. For example, it is possible that when spread on a thin surface and dried, the release of MFZ is prolonged. Though, more research is required before this is a working hypothesis.

The present study shows that foliar applied (+)ZNP(MFZ) are as effective as the formulated product, Intrepid 2F, at similar active ingredient concentrations, despite not having inert substances to increase performance. Our results appear to be consistent with other nano-entrapped insecticides, as mixed suspensions of insecticides and nanoparticles have been shown to increase susceptibility and mortality of targeted pests (Alisha and Thangapandiyan 2019). Yang et al. (2009) demonstrated that polyethylene glycol nanoparticles delayed release by nearly 70% compared to free garlic essential oil, with efficacy against *Tribolium castaneum* (Herbst) increasing after the first month of exposure. The larval weights and leaf defoliation tested in our study were similarly reduced in both (+)ZNP(MFZ) and Intrepid 2F compared to the control and unloaded (+)ZNP. It was also found that (+)ZNP(MFZ) provided greater residual concentrations of MFZ in soybean leaves under field conditions, which could suggest longer efficacy. However, the presence of higher residual insecticide indicates a conflicting challenge for integrated pest management (IPM). Integrated pest management is defined as a comprehensive decision-making approach to deal with pests, which strives to reduce pest status to tolerable levels by using methods that are effective, economically sound, and address ecological sustainability through employing management tactics so as to minimize selection pressure (Pedigo 1989, Higley and Wintersteen 1996). Selection for resistant individuals is likely to occur at a greater rate for insecticide that dissipates slowly and is less likely to develop with non-persistent pesticides since the selection pressure is lower (FAO 2012). The persistence of insecticide residues is positively correlated with a greater risk of forming resistant populations (Georghiou 1994). Balancing how to manage pests and how to control pressure for the development of resistance requires a careful assessment of the target pests, the damage they cause, and the portions of the ecosystem negatively affected by their management (Mitchell and Onstad 2014). However, exposure to longer residuals can also be valuable for pest management.

Longer exposure allows the grower continued control in the field without repeated application (Georghiou 1994, Onstad 2014, Pavan et al. 2014). Higher insecticide persistence can create asynchronous oviposition (Borchert et al. 2004), maintain coverage over the ovipositional period of pests with prolonged deposition (Teixeira et al. 2009), and retard development (Bonser et al. 2022). Nanocarriers have the potential to reduce producer input costs such as fuel and insecticide and to provide higher returns on protected crops. The challenge is that the nanocarrier-mediated longer residual time may also increase the likelihood of resistance forming. Although the results from our study indicate that after 3 DAT, MFZ levels are comparable in both (+)ZNP(MFZ) and Intrepid 2F, research suggests that nano-entrapped pesticides are released slowly (Yang et al. 2009, Hou et al. 2021). Thus, to have a longer usable life span of the nano-entrapped insecticides, an effective resistance management strategy should follow. For example, Pavan et al. (2014) suggests that long residual insecticides should be used against the last generation of the year, to not expose a subsequent larval generation to low residual levels. Georghiou (1994) details resistance management by moderation, which includes lower insecticide rates, infrequent applications, and preservation of refuge crops. Lastly, application of the same nano-entrapped insecticides should not be applied against the first generation if used against the last generation (Pavan et al. 2014).

Aguirre et al. (2013) found that MFZ concentrations of 144 ppm (mg/L) persisted on *Capsicum annuum* L. foliage for greater than 50 days under greenhouse conditions. Although there is currently no greenhouse study that evaluated (+)ZNP(MFZ) residue on soybean, our field results with 200 ppm of active ingredient, suggest that MFZ residuals persist for up to 7 days on soybean under field conditions. Residue analysis in leaf tissue found that (+)ZNP(MFZ) had higher concentrations of MFZ than Intrepid 2F at similar concentrations at 3 DAT. Although not statistically different at 7 DAT, (+)ZNP(MFZ) contained concentrations of MFZ were 46.6% higher than Intrepid 2F. Although residuals in (+)ZNP(MFZ) were higher than in Intrepid 2F at 3 DAT, this did not influence defoliation as the amount of MFZ used was lower than a recommended rate. The retuls suggests that zein biopolymeric nanoparticles may protect the active ingredient from degradation, delay the release, or provide better adhesion to the leaf tissue. Future work should focus on optimizing treatment regimens with the nanocarrier to maximize impact while minimizing dosage.
